# Biographical

**Published:** 1892-10

**Authors:** 


### Biographical.

---

### PROF. ALLEN J. SMITH, M. D.*

Allen J. Smith, M. D., Professor of Pathology and Bacteriology in the Texas Medical College (Medical Department University of Texas), at Galveston, was born in York county, Pennsylvania, in 1863. He is of German extraction and a descendant of a family of early settlers who were in Pennsylvania before the Revolutionary war, and some of whom bore arms in that memorable struggle for independence. He received his early education in his native county and his collegiate education at Gettysburg —taking first honor and the degree of A. B. in 1883, and the degree of A. M. in 1886. He attended the courses in the Medical Department of the University of Pennsylvania, and in 1886 graduated amongst the first honor men, receiving his degree of M. D, and carrying off the *Medical News* prize and the anatomical prize. Since graduating, Dr. Smith has held the following positions: Resident Physician in Philadelphia Hospital, 1886; Salaried Resident Physician (male department) of the Insane Wards in same in 1887; Assistant Demonstrator of Pathology in University Pennsylvania in 1887; Master in Chemistry in Medical Institute (allied to University of Pennsylvania) in 1887; Physician to Dispensatory for Diseases of Children in University Hospital, same year, and chief of same in 1891; Acting Pathologist to Blockley Hospital for part of 1890; Master in Pathology in Medical Institute in 1889; Pathologist to Presbyterian Hospital in 1891; Assistant Pathologist to University Hospital, 1890-91; Pathologist and Visiting Physician to State Insane Asylum at Norristown, Pa., in 1891; Lectnrer on Urinology in University of Pennsylvania, 1889; Editor (part of the time with Dr. James Tyson) of articles on Urinalysis, Diseases of Kidneys, etc., and Diabetes, in the Annual of Universal Medical Sciences for 1889, 1890, 1891-2 (four years); Editor of "Notes on Pathology for students of University of Pennsylvania," published in 1892; Editor of Naphey's Modern Therapeutics, 2 vols., 1892; Editor of Vol. XV. Transactions of Philadelphia Pathological Society, 1891; Editor Pathological section in Lippincott's International Medical Journal; Recorder of Pathological Society of Philadelphia, 1890-91.

In addition to the above mentioned positions, Dr. Smith has filled a number of other positions, such as Microscopist to the University Commission on Koch's Tuberculosis Cure, etc., and was in active work when elected to the honorable and responsible position he now holds in the University of Texas Medical College.

Few men of his age have been more actively and honorably

---

*In our souvenir edition, July, 1892, in which were published the biography and portraits of the members of the faculty of the Texas Medical College (Medical Department University of Texas), this notice should have appeared. It was accidentally omitted and is here published to complete the set.—ED.

employed or attained to such eminence in a profession notably crowded with able men; still, Dr. Smith has found time to contribute to the various medical journals; and his writings may be found in most of the Philadelphia journals.

The Review, in this issue of DANIEL'S TEXAS MEDICAL JOURNAL, of Cholera in India, was written by Dr. Smith, and the JOURNAL is pleased to announce that he will contribute to its pages during the coming year, a part of the rich clinical material falling under his observation as Microscopist, Pathologist and Bacteriologist in the Texas Medical College, he having consented to become one of the assoiate editors of this journal.

Dr. Smith is married, and his family reside with him in Galveston. The portrait of him which appeared in our July number hardly did him justice. The doctor is blessed with a strong physical organization; none other could stand the tremendous drafts made upon his nerve force by such an active and hard worked brain.

---

Naphey's Therapeutics.—In the last number of the JOURNAL, September, '92, we published a notice of the new edition of *Naphey's Therapuetics*, just issued from the press of P. Blakiston, Son & Co., Phila. (see page 116), edited by Drs. Allen J. Smith and J. Aubrey Davis. By some provoking oversight, we omitted to give the name of the *publishers.* We extend to them now a sincere apology, and trust they will make amends.

We were struck by the name "Allen J. Smith," and upon inquiry we learn that Dr. Allen J. Smith, of Philadelphia, the editor, is Prof. Allen J. Smith, now of the Chair of Pathology and Bacteriology in the Medical Department, University of Texas. We did not know it, however, before the appearance of our September number. It is mentioned here and now, in justice to Dr. Smith, and because we hope and believe that it will cause the Texas students to take more interest in the book than they would probably do if it were edited by any other "Dr. Smith." It serves to show that the Regents of our Texas University recognize talent, and spare no expense in bringing it to the Texas Medical College.

